# Non-inferiority of short-term urethral catheterization following fistula repair surgery: study protocol for a randomized controlled trial

**DOI:** 10.1186/1472-6874-12-5

**Published:** 2012-03-20

**Authors:** Mark A Barone, Vera Frajzyngier, Steven Arrowsmith, Joseph Ruminjo, Armando Seuc, Evelyn Landry, Karen Beattie, Thierno Hamidou Barry, Alyona Lewis, Mulu Muleta, Dolorès Nembunzu, Robert Olupot, Ileogben Sunday-Adeoye, Weston Khisa Wakasiaka, Mariana Widmer, A Metin Gülmezoglu

**Affiliations:** 1 Fistula Care Project, EngenderHealth, 440 Ninth Avenue, 13th Floor, New York, NY 10001, USA; 2 Fistula Consulting LLC, 452 Union Ave. SE, Grand Rapids, MI 49503, USA; 3 World Health Organization, Department of Reproductive Health and Research, 1211 Geneva 27, Geneva, Switzerland; 4 L'Hôpital Préfectoral de Kissidougou, Kissidougou, Guinea; 5 Aberdeen Women's Centre, PO Box 416, Freetown, Sierra Leone; 6 Gondar University Hospital, Fistula Unit, PO Box 196, Gondar, Ethiopia; 7 Hôpital Saint Joseph, 15ème Rue, Boulevard Lumumba, Limete Résidentiel, Kinshasa, Democratic Republic of Congo; 8 Kagando Hospital, Kasese District, Uganda; 9 National Obstetric Fistula Centre Abakaliki, 1 Water Works Street, Abakaliki, Ebonyi State, Nigeria; 10 Kenyatta National Hospital, PO Box 20723-00202, Nairobi, Kenya

**Keywords:** Vaginal fistula, Catheter, Non-inferiority randomized controlled trial, Surgery

## Abstract

**Background:**

A vaginal fistula is a devastating condition, affecting an estimated 2 million girls and women across Africa and Asia. There are numerous challenges associated with providing fistula repair services in developing countries, including limited availability of operating rooms, equipment, surgeons with specialized skills, and funding from local or international donors to support surgeries and subsequent post-operative care. Finding ways of providing services in a more efficient and cost-effective manner, without compromising surgical outcomes and the overall health of the patient, is paramount. Shortening the duration of urethral catheterization following fistula repair surgery would increase treatment capacity, lower costs of services, and potentially lower risk of healthcare-associated infections among fistula patients. There is a lack of empirical evidence supporting any particular length of time for urethral catheterization following fistula repair surgery. This study will examine whether short-term (7 day) urethral catheterization is not worse by more than a minimal relevant difference to longer-term (14 day) urethral catheterization in terms of incidence of fistula repair breakdown among women with simple fistula presenting at study sites for fistula repair service.

**Methods/Design:**

This study is a facility-based, multicenter, non-inferiority randomized controlled trial (RCT) comparing the new proposed short-term (7 day) urethral catheterization to longer-term (14 day) urethral catheterization in terms of predicting fistula repair breakdown. The primary outcome is fistula repair breakdown up to three months following fistula repair surgery as assessed by a urinary dye test. Secondary outcomes will include repair breakdown one week following catheter removal, intermittent catheterization due to urinary retention and the occurrence of septic or febrile episodes, prolonged hospitalization for medical reasons, catheter blockage, and self-reported residual incontinence. This trial will be conducted among 512 women with simple fistula presenting at 8 study sites for fistula repair surgery over the course of 24 months at each site.

**Discussion:**

If no major safety issues are identified, the data from this trial may facilitate adoption of short-term urethral catheterization following repair of simple fistula in sub-Saharan Africa and Asia.

**Trial registration:**

ClinicalTrials.gov Identifier NCT01428830.

## Background

A vaginal fistula is a devastating condition, and while true prevalence is unknown, it is estimated to affect 2 million girls and women across Africa and Asia. The primary cause of a vaginal fistula is prolonged obstructed labor: the fetus's head compresses the soft tissues of the bladder, vagina and rectum against the woman's pelvis, cutting off blood supply to the tissue, causing the tissue to die and slough away. The result is an abnormal opening between the vagina and bladder, or between the vagina and rectum, or both, and either urinary and/or fecal incontinence.

Approximately 80-95% of vaginal fistula can be closed surgically [1]; however, the provision of fistula repair services in developing countries is not without challenges. Specialized training and skills are necessary, especially to handle more complex cases, and this limits the availability of services. Women with fistula are predominantly poor and from rural areas, and often cannot pay for surgery or transport to a service site. Thus, fistula repair services must be provided free of charge. Fistula services are few and far between in Africa and Asia: the availability of services depends not only on the availability and motivation of surgeons with specialized skills, but also the availability of operating rooms, equipment, and funding from local or international donors to support both surgeries and lengthy post-operative care. In most contexts, the need for repair services exceeds the available human and infrastructural capacity. Moreover, the prolonged bladder catheterization that is frequently employed after surgery translates into a need for longer hospitalization, more intensive nursing care, and increased costs, leading to decreased capacity for treating other patients. Longer duration catheterization may also be associated with increased risk of healthcare-associated infection. Reducing the duration of bladder catheterization, provided no compromise in surgical outcomes and patient health, has important implications for the delivery of fistula care and treatment services in low resource settings.

Bladder catheterization practices following fistula repair and other pelvic surgery vary. Publications about fistula surgery often state that bladder drainage through indwelling urethral catheterization should continue between 10 and 14 days post-operatively [2,3]. However, in both fistula repair [4] and other types of gynecological surgery [5], the duration of bladder drainage via indwelling urinary catheter is primarily based on custom rather than empirical research, and the duration of drainage varies substantially. A recent survey of 40 fistula surgeons conducted by Arrowsmith and colleagues [6] found that catheterization durations following fistula repair surgery ranged from 5 to 21 days, with 13% of surgeons reporting they catheterize women for 8 days or less following surgical repair of simple fistula. Results from a multi-country prospective cohort study examining predictors of repair outcomes recently completed by Fistula Care/USAID also found a wide distribution in post-repair duration of urethral catheterization among 1274 women. The median duration of catheterization was 21 days, with an interquartile range (IQR) of 14-27 days [7]. A wide range has also been found for duration of catheterization following colovesical fistula repair. A retrospective review of data at Massachusetts General Hospital conducted by de Moya and colleagues found that duration of catheterization following colovesical fistula repair secondary to diverticulitis ranged from 3-42 days [8]. While colovesical fistula repairs differ considerably from vaginal fistula repairs, these findings nonetheless indicate that not only is shorter-term catheterization currently being implemented for different types of bladder surgery, but that it may be a feasible alternative to longer term catheterization following the repair of simple urinary fistula in women.

Short-term urethral catheterization may in fact pose no additional risk to patients in terms of repair prognosis. One purpose of post-repair urethral catheterization is to provide an opportunity for adequate tensile strength to develop so that bladder distension does not disrupt the healing wound. The presumption behind prolonged catheterization is that the bladder heals better "at rest" (i.e. when it is not filling and emptying); however, there is little evidence to support this. For non-contaminated wound healing in general, the critical period involving granulation and neovascularization peaks at 5 days and inflammation is over in 1 week [9], with matrix deposition and cell proliferation continue until at least 30 days. Tensile strength increases rapidly during the first 5 days, although final tensile strength is not reached until over 100 days. A recent study evaluating the effects of long-term catheterization on extracellular matrix (ECM) biological scaffold remodeling following partial cystectomy in canines, found that early bladder filling (i.e. shorter duration of catheterization) mediated a constructive remodeling response [10]. While biologic scaffolds composed of ECM are a cutting edge innovation not feasible for fistula repair in developing countries, these results suggest that removing the catheter early and allowing the bladder to begin filling and emptying, may be beneficial, rather than harmful, to bladder healing. Nonetheless, no basic physiologic studies on the dynamics of wound healing in the bladder after fistula repair have been published to date and it is possible that the wound healing process following fistula repair may be more prolonged than noted above given the contaminated and chronic nature of most vaginal fistula.

Given the potential benefits of short-term urethral catheterization, and the fact that it is currently being practiced by some fistula surgeons, empirical evidence is needed to determine the non-inferiority of short-term urethral catheterization compared to longer-term urethral catheterization. The primary objective of this study is thus to examine whether short-term (7 day) urethral catheterization is not worse by more than a minimal relevant difference to longer-term (14 day) urethral catheterization in terms of incidence of fistula repair breakdown among women with simple fistula.

## Methods/Design

**Figure 1:**
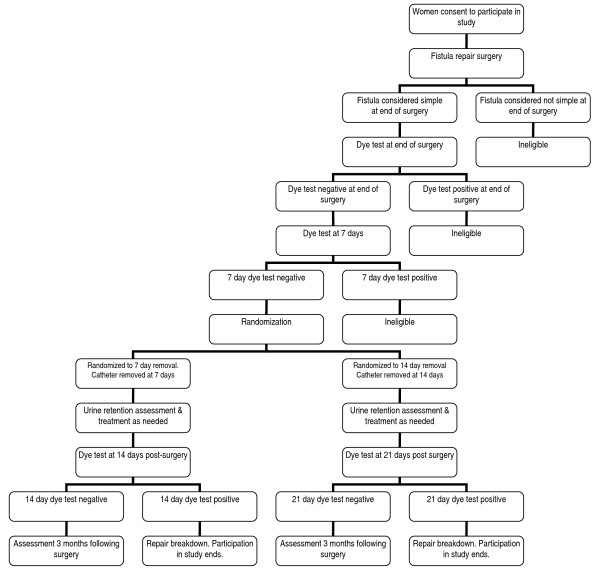
**Trial profile**. Women who consent to participate in the study may be determined ineligible prior to randomization: (1) at the time of surgery if the surgeon determines that the fistula is 'not simple': (2) at the end of surgery if the fistula is not closed based on dye test results; and (3) 7 days after surgery if the fistula is not closed based on dye test results. Seven days after surgery, women with a closed fistula will be randomized to catheter removal on that day (i.e. the 7-day removal group) or to have the catheter kept in place for an additional 7 days (i.e. the 14-day removal group). Women will remain at the study site for an additional 7 days after catheter removal. On days 1, 3 and 7 after catheter removal, urine retention will be assessed and intermittent catheterization employed as needed. Women with a positive urinary dye test at 7 days following catheter removal will be classified as having experienced a repair breakdown and their participation in the study will be completed. Remaining women will be asked to return for a follow-up visit 3 months after the date of surgery.

This will be a non-inferiority randomized controlled trial (RCT) comparing the new proposed short-term (7 day) urethral catheterization to longer-term (14 day) urethral catheterization in terms of predicting fistula repair breakdown, measured 3 months following fistula repair surgery. This study will be conducted among 512 women with simple fistula presenting at eight study sites for fistula repair surgery (Table 1). Figure 1 depicts the flow of study participants.

**Table 1:** Study sites

Country	Site
Democratic Republic of Congo	Hôpital Saint Joseph de Kinshasa

Ethiopia	Gondar University Hospital Fistula Unit

Guinea	Prefectural Hospital of Kissidougou

Kenya	Kenyatta National Hospital

Niger	Maternité centrale de Zinder

Nigeria	National Obstetric Fistula Centre Abakaliki

Sierra Leone	Aberdeen Women's Centre

Uganda	Kagando Hospital

### Intervention

The study intervention will entail removal of the urethral catheter at 7 days after surgery in the intervention arm and 14 days after surgery in the control arm. While 7 and 14 days are the target days for urethral catheter removal in the two groups, should it be necessary to remove the catheter either one day earlier or one day later (e.g. holiday, surgeon unavailable) in either group (i.e. 6-8 days or 13-15 days) these women will still be considered as compliant with the group assignment. Each patient will receive the same type of catheter, and with the exception of the timing of catheter removal, all other procedures related to the removal of the catheter will remain the same across both study arms. No drugs or new devices will be examined as part of this study.

### Outcome measures

The primary study endpoint is fistula repair breakdown any time after day 7 after urethral catheter removal up to three months following surgery. Repair breakdown will be assessed using a dye test; a urinary catheter will be inserted into the bladder, saline colored with dye will be introduced into the bladder via the catheter, and the suture line will be checked for leaks.

Secondary outcomes will include: repair breakdown one week following indwelling urethral catheter removal, the need for intermittent catheterization to manage urinary retention, and the occurrence of septic or febrile episodes, prolonged hospitalization, catheter blockage, and self-reported residual incontinence. Prolonged hospitalization will be defined as a stay at the facility beyond one week following initial catheter removal for medical reasons.

### Study population

All women who present at study sites for fistula repair surgery will be potentially eligible. Women will be randomized if:

• they have a "simple" fistula, based on the surgeon's perception during surgery

• they have a closed fistula at completion of surgery

• they have a closed fistula 7 days after surgery

• they understand study procedures and requirements

• they agree to return to the facility for one follow-up visit three months after the date of surgery

• they provide informed consent to participate in the study

• they have no contraindications precluding their participation

Women will be excluded if they have a fistula that is:

• determined to be "not simple"

• radiation-induced, associated with cancer or due to lymphogranuloma venereum

• not closed immediately after surgery or 7 days after surgery

### Generation of allocation sequence

Randomization will be done using permuted blocks within site to ensure similarity of groups with regard to potential confounding factors that might influence repair outcomes within a particular study site. The random allocation sequence will be generated centrally at the World Health Organization (WHO) Headquarters using computer generated random numbers. Randomization will be to two groups and stratified by site. Blocking with randomly varying groups will be used to restrict randomization within the strata. Each site will recruit 64 participants.

### Random allocation technique and allocation concealment

Allocation of the random generated sequence will be by consecutively numbered envelopes. Allocation concealment will be achieved by using sealed opaque envelopes. Allocation will take place 7 days after fistula repair surgery.

### Rationale for the non-inferiority hypothesis and for sample size estimation

The research question of interest is whether short-term catheterization is not worse by more than a minimal relevant difference than longer-term catheterization in terms of achieving fistula closure. This question lends itself to a non-inferiority design.

The choice of a non-inferiority margin, i.e. the smallest clinical difference that is acceptable between the two treatments, is based on a combination of clinical judgment and statistical reasoning. Because there are no data from previous trials to help define the clinical difference between treatments, we have relied on our own and outside experts' clinical judgment to determine that a margin of inferiority of 10% is an irrelevant small difference. In other words, if the two-sided 95% confidence interval (95% CI) for the difference in fistula repair breakdown rates ("7-day" minus "14-day") lies fully to the left of the 10% non-inferiority margin, we will have proved non-inferiority of the "7-day" procedure at the level of significance *α *= 0.025; superiority (as a bonus) will be demonstrated at the level of significance *α *= 0.05 if the two-sided 95% CI lies fully to the left of 0.

Analyses were conducted using preliminary data from the Fistula Care/USAID prospective cohort study examining fistula repair outcomes in order to determine the probability of successful closure in women with simple fistula catheterized for longer periods of time (i.e. the equivalent of the "standard" treatment group in the study outlined here). Among the women with simple repairs in the prospective study for whom follow-up data were available (n = 145), 87% had a closed fistula at 3 months follow up. Thus, we believe that it is reasonable to expect the failure rate (e.g. proportion of fistula that are not closed) to be between 10 to 15%.

Assuming 13% failure rate in the control group, non-inferiority will be demonstrated within the margin of 10% at a one-sided significance level of 0.025 and a power of 80% (calculated when failure rates in both arms are the same), with a sample size of 177 per arm (354 women in total). Adjusting by 20% for loss to follow-up and 10% for protocol violations and withdrawals, this would result in a sample size of 507 women. Each site will randomize 64 women, for a total sample size of 512.

### Type of data and collection procedure

For all women who have consented to participate, data will be collected, at hospital level, at three time points using case report forms (CRFs) developed for the study: i) prior to surgery, detailed information on socio-demographic characteristics of the women, duration of the fistula, previous attempts at repairing the fistula and pre-operative care procedures will be recorded; ii) at the time of surgery, a detailed clinical examination will be conducted to assess the characteristics of the fistula, and to allow the surgeon to determine if he/she would consider the fistula "simple" or not. For all participants, detailed information will be recorded on the anatomical and clinical characteristics of the fistula(s), including the presence of scarring, location and type of the fistula and length and width of the fistula; iii) post-surgery, at 1, 3, 7 and 90 days after surgery. Completed CRFs will be sent periodically to the data management centre at the WHO in Geneva for data entry.

### Analysis plan

The primary outcome of the trial is repair breakdown at 3 months or earlier in an intent-to-treat (ITT) analysis. The primary analysis will be conducted for the ITT population. The ITT population would include patients for whom the protocol is violated (i.e. patients who are randomized to short-term catheterization but are in fact catheterized for longer durations, or patients who are randomized to the long-term catheterization group but are in fact catheterized for only 7 days) and withdrawals.

**Figure 2:**
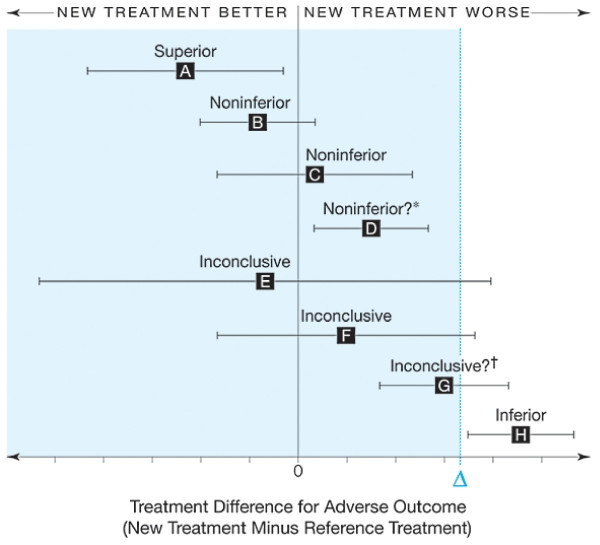
**Interpreting results of non-inferiority trials **[11]. Footnotes for Figure 2. *This CI indicates noninferiority in the sense that it does not include Δ, but the new treatment is significantly worse than the standard. Such a result is unlikely because it would require a very large sample size. †This CI is inconclusive in that it is still plausible that the true treatment difference is less than Δ, but the new treatment is significantly worse than the standard.

Unlike a superiority trial, where the treatment effect is the primary parameter of interest, the interpretation of a non-inferiority trial's results depends on the location of the CI for the effect of the experimental treatment relative to the margin of non-inferiority (Δ) and a null effect. A range of possible scenarios are depicted in Figure 2, where error bars indicate 2-sided 95% CIs, and the tinted area indicates the zone of inferiority. Thus, primary analyses will be interpreted as follows: if the whole 95% CI lies to the right of the non-inferiority margin of 10% (scenario H), the experimental intervention will be declared "inferior." If the whole 95% CI lies below the non-inferiority margin, the intervention will be considered to be non-inferior to the standard treatment (scenarios B-D). If the 95% CI includes the non-inferiority margin Δ (scenarios E, F and G) study results will be deemed inconclusive. Finally, in scenario A, since the 95% CI lies completely to the left of zero, the new treatment may be considered superior to the standard treatment [11].

Once non-inferiority is demonstrated, it is acceptable to then test the hypothesis that the new treatment is superior to the active control, with a significance level defined a priori and with an ITT analysis [12].

### Interim data analysis

A Data Safety and Monitoring Board (DSMB) with no direct involvement in the trial will be appointed. The role of the DSMB will be to deal with any ethical issues that may arise while the trial is in progress, and to scrutinize an interim analysis. An interim analysis will be conducted to be reviewed by the DSMB after one-third of the study participants have returned for the three-month follow-up visit. At the time of the interim analyses, event and recruitment rates will be provided to the DSMB by unmasked treatment group.

### Stopping the trial

The DSMB will be asked to give advice regarding stopping the trial if they have proof beyond doubt of an important advantage or disadvantage for one of the treatment groups, and they consider that the results are likely to affect clinical practice. For the outcomes of the trial, the following stopping guidelines are proposed for the DSMB:

(1) At the time of the interim analysis, the DSMB may recommend stopping the study or temporarily halting recruitment if there are significantly more repair breakdowns in one catheterization group compared to the other; a difference between the two treatment arms will be considered significant if the p value for the difference is less than 0.001.

(2) In the event that the interim analysis shows notably more adverse events in either study arm, the DSMB may recommend stopping the study or temporarily halting recruitment.

(3) The DSMB may also recommend stopping the study, temporarily halting recruitment or adjusting study sites if it seems that recruitment is not proceeding at rates that will allow the study to reach its target sample size in a reasonable timeframe.

It should be noted that if there appears to be an unexpectedly high number of repair breakdowns among short-term catheterization cases compared to long-term catheterization cases either as reported by study site staff or the study monitor, or based on the interim analysis, any one of the study Institutional Review Boards (IRBs) may also temporarily or permanently halt the study at any time.

If the study is stopped temporarily or permanently for any reason, follow-up of women already enrolled will continue as originally scheduled and all women already enrolled will receive continued care, appropriate to their clinical condition and circumstances, in line with each sites standard practice.

### Duration of project

It is anticipated that the recruitment into the trial and all follow-up of participants can be completed in approximately 2 years. Recruitment will begin in December 2011 after trial procedures have been tested, study staff trained and materials have been distributed to the study sites.

## Discussion

If no major safety issues are identified, the data from this trial may facilitate adoption of short-term catheterization following repair of simple fistula in various countries in sub-Saharan Africa and Asia. The adoption of short-term catheterization will have important implications for service delivery, and potentially, patient health outcomes. For instance, Nardos and colleagues calculated the implications of urethral catheterization duration at the Addis Ababa Fistula Hospital, where approximately 1200 fistula repairs are performed annually. They reported that assuming no compromise in patient outcomes, a four day reduction in postoperative hospitalization due to early bladder catheter removal (10 vs. 14 days) would allow the number of patients who could receive surgical care to be increased by 20% [9]. In light of the wide range of catheterization durations practiced in sub-Saharan Africa and Asia [6,7], the potential benefit associated with short-term catheterization with regard to increased access to surgery for women with obstetric fistula may be significantly greater. In addition, short-term duration of urethral catheterization may also decrease risk of urinary tract infection (UTI). For instance, a recent Cochrane review of urinary catheterization following urogenital surgery in adults examined seven trials which compared shorter postoperative duration of catheter use to longer duration; these trials suggested that shorter-term catheterization was associated with fewer UTIs [13]. In sum, short-term urethral catheterization has the potential to reduce hospital stays for women, thus freeing bed space, reducing costs per patient, and allowing for a greater number of patients to receive clinical care. It may also have implications for the probability of infection, and possibly sepsis, following surgery. Irrespective of study outcome, the results of this study will be disseminated in at least one workshop/forum attended by fistula surgeons, regional/local meetings, and at least one publication in a peer-reviewed journal.

## Technical and Ethical Approvals

The protocol received technical and ethical approval from the WHO Research Project Review Panel (RP2) and Research Ethics Review Committee, respectively. Ethical approval will be obtained from appropriate national and institutional ethical review bodies as applicable for each study site. Recruitment of participants at any given site will not begin until all the necessary local ethical approvals have been obtained.

## Competing interests

The authors declare that they have no competing interests.

## Authors' contributions

JR and SA initially conceived the project. MAB and VF drafted the study protocol and contributed to the design of the study. SA, JR, AS, EL, KB, THB, AL, MM, DN, RO, ISA, WKW, MW, AMG contributed to the design of the study and provided critical review and input to the study protocol. All authors provided a critical review and final approval of the manuscript.

## Pre-publication history

The pre-publication history for this paper can be accessed here:

http://www.biomedcentral.com/1472-6874/12/5/prepub
